# The promise and challenges of multi-cancer early detection assays for reducing cancer disparities

**DOI:** 10.3389/fonc.2024.1305843

**Published:** 2024-03-08

**Authors:** Cheryl L. Thompson, Monica L. Baskin

**Affiliations:** ^1^ Department of Public Health Sciences, Penn State Cancer Institute, Pennsylvania State University College of Medicine, Hershey, PA, United States; ^2^ University of Pittsburgh Medical Center, Hillman Cancer Center, University of Pittsburgh, Pittsburgh, PA, United States

**Keywords:** cancer, screening, disparities, multi-cancer detection assays, health equity

## Abstract

Since improvements in cancer screening, diagnosis, and therapeutics, cancer disparities have existed. Marginalized populations (e.g., racial and ethnic minorities, sexual and gender minorities, lower-income individuals, those living in rural areas, and persons living with disabilities) have worse cancer-related outcomes. Early detection of cancer substantially improves outcomes, yet uptake of recommended cancer screenings varies widely. Multi-cancer early detection (MCED) tests use biomarkers in the blood to detect two or more cancers in a single assay. These assays show potential for population screening for some cancers—including those disproportionally affecting marginalized communities. MCEDs may also reduce access barriers to early detection, a primary factor in cancer-related outcome disparities. However, for the promise of MCEDs to be realized, during their development and testing, we are obligated to be cautious to design them in a way that reduces the myriad of structural, systematic, and personal barriers contributing to disparities. Further, they must not create new barriers. Population studies and clinical trials should include diverse populations, and tests must work equally well in all populations. The tests must be affordable. It is critical that we establish trust within marginalized communities, the healthcare system, and the MCED tests themselves. Tests should be expected to have high specificity, as a positive MCED finding will trigger additional, oftentimes invasive and expensive, imaging or other diagnosis tests and/or biopsies. Finally, there should be a way to help all individuals with a positive test to navigate the system for follow-up diagnostics and treatment, if warranted, that is accessible to all.

## 1 Introduction

Since the development of cancer therapeutics and advances in cancer detection, diagnosis, and prevention, cancer disparities have existed. Marginalized populations, which include racial and ethnic minorities, sexual and gender minorities, individuals with lower incomes, those living in rural areas, and persons living with disabilities, have higher mortality and other cancer-related health outcomes. For example, Black women have a 40% higher breast cancer mortality compared to White women (1), and racial gaps in cancer mortality exist even when accounting for socioeconomic status (2). As another example, individuals living in rural counties have lower cancer incidence but higher mortality for most cancers, and this gap is increasing (3).

## 2 Disparities in cancer screening

Cancer outcomes improve substantially with early detection, yet uptake of recommended cancer screenings varies widely. Current U.S. Preventive Services Task Force (USPSTF) recommendations for individuals at average risk include regular screenings for breast, colorectal, and cervical cancers. The USPSTF further recommends lung cancer screening for current and former smokers with a 30-year pack-year history of smoking. For prostate cancer, informed decision-making between an individual and clinician accounting for family history and African-American race is recommended.

Rates of breast cancer screening overall are similar among women from different racial and ethnic groups. However, there are substantial variations in both uptake of newer, more powerful, imaging technologies (4) and follow-up after positive findings (5). For example, in one study, Black women were much less likely (44%) than White women (61%) to undergo digital breast tomosynthesis in conjunction with mammography (4). Another study noted that the average days to diagnostic follow-up after an abnormal mammogram were notably higher for Hispanic women (21 days) compared with non-Hispanic White women (14 days). Further, substantially lower screening prevalence of breast, cervical, and colorectal cancers was noted among individuals with household incomes of less than $50,000 (6). Racial minorities and socioeconomically disadvantaged individuals are much less likely to obtain lung cancer screening (7). For example, a in central North Carolina showed that almost 93% of all individuals screened for lung cancer were White, whereas there were only 61.5% of the smokers in the county (8). Further, colorectal cancer screening rates are significantly lower among Hispanic and other racial/ethnic minority groups, and the implementation of the Affordable Care Act did not substantially change these disparities (9).

Reasons for disparities in screening are numerous and are structural, systematic, and personal. Structural barriers include limited access to screening facilities and transportation, screening and treatment costs, and limited insurance coverage (10). System barriers include racism, lack of workforce diversity, limited research incorporating diverse populations, and inequitable distribution of high-quality cancer care (e.g., new targeted and immune therapies) (10, 11). Personal barriers include medical literacy, misinformation, and mistrust of the healthcare system (12) along with discomfort with screening procedures. It is important for us to understand and attempt to mitigate as many of these reasons for screening disparities as possible, as new technology is being developed and implemented.

## 3 Multi-cancer early detection tests for cancer screening

Multi-cancer early detection (MCED) tests use a variety of biomarkers in the blood to detect two or more cancers in a single assay. There are many different technologies being used to develop these tests, including DNA methylation or circulating proteins (13). Further, these tests are being developed in numerous fluids, including whole blood, plasma, and urine. Some provide output that just estimates the likelihood of cancer, some predict one or more most likely organ sites, and some test for a subset of specific cancers. MCED tests are still currently in development and refinement, and large-scale trials for widespread screening in an asymptomatic population have yet to be completed. However, many of these assays show promise as being the first potential population screening tests for some cancers—including cancers that disproportionally affect minority communities, such as stomach cancer in Black and American Indian or Alaska Native populations.

MCEDs have the potential to reduce many of the barriers to screening mentioned above and thus reduce screening disparities. As blood-based tests, they do not require large imaging machines and the upfront costs associated with implementing those in low-resource areas. A blood sample can be taken at your local clinic and does not involve taking hours to go to the large hospital This convenience may be particularly relevant for rural residents, those without personal transportation, and/or persons who would experience hardship in taking substantial time off from work/home responsibilities for screening or screening preparation (e.g., bowel preparation for colonoscopy). In fact, home self-sampling has shown promise for increasing screening rates in medically underserved populations (14, 15). If an MCED can be performed with a small finger prick of blood, self-sampling for MCED testing could potentially have an even larger impact in harder-to-reach communities.

## 4 Recommendations for MCED development and testing

**Figure 1:**
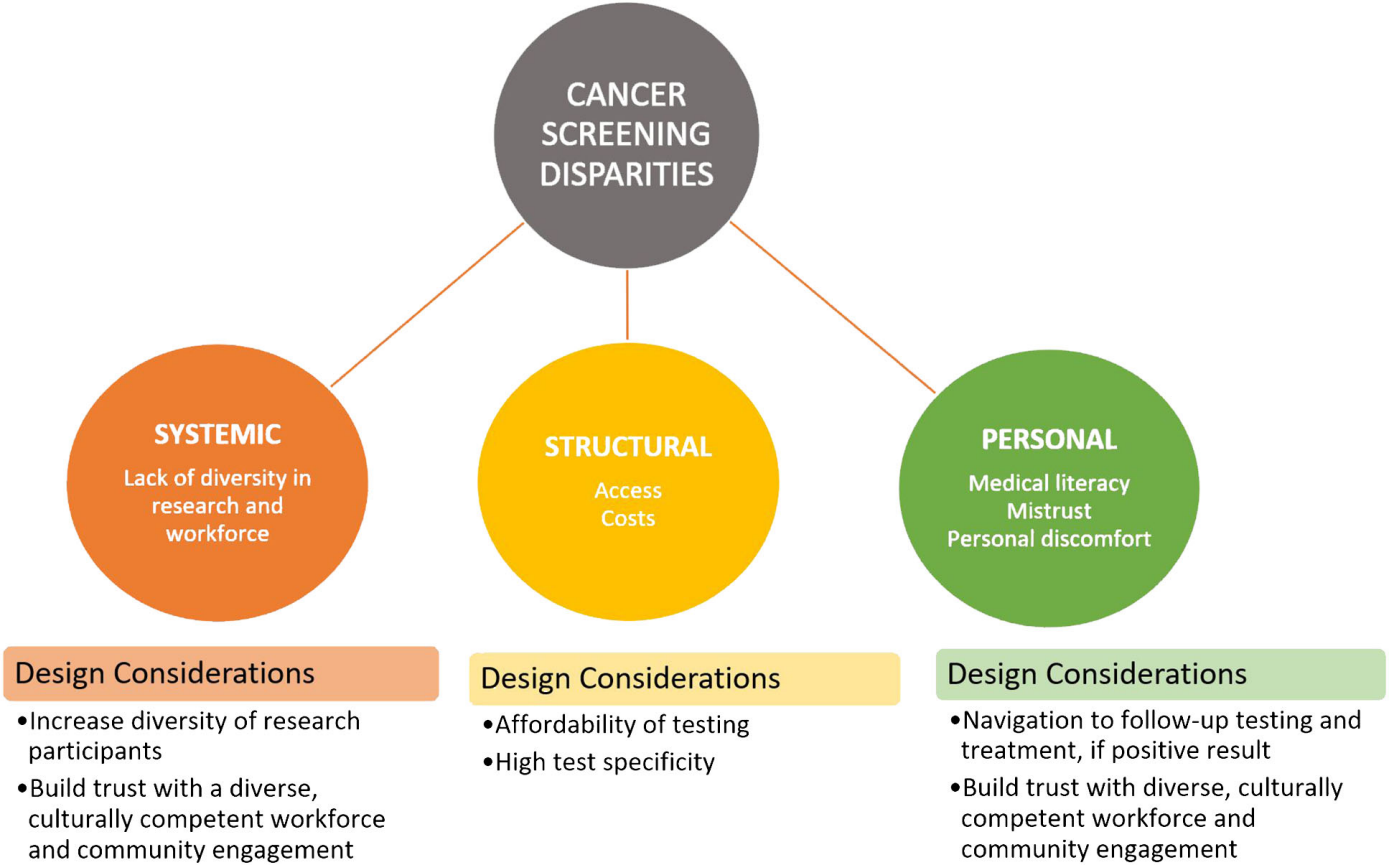
Key considerations for multi-cancer early detection assay developments.

For the promise of MCEDs to reduce these barriers to be realized, during their development and testing, we must be cautious to design them in a way that ameliorates these barriers rather than exacerbate them or create new ones. Therefore, we present a number of factors for consideration in future MCED research (**Figure 1**).

### 4.1 Population studies and clinical trials must include diverse populations

It is critical that our research on the safety, efficacy, and acceptability of these tests includes individuals from diverse groups. These groups include different racial and ethnic backgrounds; residences; physical, mental, and language abilities; and socioeconomic status. Further, we should make sure we have a particular focus on capturing those for whom we know are experiencing cancer disparities. Clinical trials are often a critical option for improved patient care and health outcomes; however, overall participation by minority populations is low (estimated at 2%–8% of adults with cancer) and not reflective of the racial/ethnic demographic population of the USA. For example, recent data indicate that cancer therapeutic trials are made up of only 4%–6% Black and 3%–6% Hispanic patients even though they represent 15% and 13% of cancer patients, respectively (16). As such, the need for representation in research trials is to ensure that interventions work equally well in all populations. When certain populations are not well studied, the generalizability of the findings to these communities is unclear, and the benefit of the research may only help well-represented communities.

### 4.2 Tests must be affordable

To our knowledge, there are currently two MCED tests on the market. OneTest™ is marketed directly to consumers for $189 plus shipping but is not particularly sensitive or specific (17). Galleri^®^, ordered through medical providers, costs almost $1,000 (18). Neither test is currently covered by commercial or government health insurance, making it less likely for those more likely to experience disparities (i.e., lower income) to benefit. Ensuring affordability and therefore access to this new technology also has the potential to reduce healthcare costs, as MCEDs have the potential to shift cancer diagnoses to earlier stages (19).

### 4.3 We must establish trust in the system and MCED tests

Decades of historical trauma resulting from unethical medical research practices has fueled mistrust in the medical system. The current rampant spread of misinformation along with mistrust has combined to foster a culture where members of marginalized communities are more reluctant to adopt new interventions or technologies. An intentional effort to build and maintain trust and reduce the impacts of medical mistrust on patient outcomes is needed. Interventions should follow established models of community-academic engagement (20), increase diversity in the biomedical workforce (21), enhance medical school curricula (22), and improve patient-centered communication (23).

### 4.4 The tests must have high specificity

MCEDs are intended as a cancer screening test for people without symptoms. As such, a positive finding will trigger additional, oftentimes invasive and expensive, imaging or other diagnostic tests and/or biopsies. If a positive finding exists for multiple cancers, there potentially would be multiple sets of follow-ups. If this follow-up detects early-stage cancer, we would generally consider this a success, as more treatment options are available with lower associated medical costs than later-stage diagnosis. However, if the follow-up does not detect cancer, it creates unnecessary testing, burden, worry, and cost for the patient and healthcare system. The state of currently available MCEDs varies with respect to the specificity of the test, and thus, there is reason for caution in recommendations of this technology for the general public. Large-scale randomized trials with diverse populations are needed to establish an evidence base for the widespread use of this screening tool.

### 4.5 There must be a way to help all individuals navigate the system to quality follow-up diagnostics and, if confirmed, treatment when positive results arise

MCEDs are used to detect signals or biomarkers that are released by cancer cells in the blood. Positive findings or signals from these tests need to be followed up with diagnostic testing. Assurance of equitable and timely follow-up implemented *via* a direct-to-consumer test is unclear but must be addressed. Patient navigation programs targeting historically marginalized populations have demonstrated effectiveness in cancer screening (24) and follow-up (25). Even for negative tests, patients may be unclear as to what further cancer screening they may need, or when to have the MCED test again. Tests available clinically should consider patient navigation or other evidence-based ways to increase cancer screening follow-up.

## 5 Discussion

MCED tests for cancer screening are an exciting new development in cancer control. Their promise for the early detection of cancers for which currently no screening methods are available and to make screening easier for all can help reduce disparities in screening. However, we must use caution as we develop these tests and not continue to develop technology that only benefits privileged groups. It is critical that we recruit diverse populations for trials so that we can ensure the tests work well in all populations. Then, we must understand how implementing them in real-world conditions works and how they can be scaled in ways that increase screening uptake in areas with low screening rates. This effective research, particularly in underserved populations, will be critical to realizing the promise of MCEDs to reduce cancer disparities. Here, we offered five key, albeit complex and not easy, things that we believe must be taken into consideration early in the MCED development process to ensure that uptake is performed in a more equitable way. We are excited about the potential of MCED testing and, if performed right, their ability to help us close the gaps in cancer screening. We encourage funding agencies to sponsor further research on using MCED testing to reduce cancer disparities.

## Data availability statement

The original contributions presented in the study are included in the article/supplementary material. Further inquiries can be directed to the corresponding author.

## Author contributions

CT: Conceptualization, Writing – original draft, Writing – review & editing. MB: Conceptualization, Writing – original draft, Writing – review & editing.
